# The impact of memantine in combination with acetylcholinesterase inhibitors on admission of patients with Alzheimer’s disease to nursing homes: cost-effectiveness analysis in France

**DOI:** 10.1007/s10198-013-0523-y

**Published:** 2013-08-09

**Authors:** Jacques Touchon, Jean Lachaine, Catherine Beauchemin, Anna Granghaud, Benoit Rive, Sébastien Bineau

**Affiliations:** 1 Faculty of Medicine, University of Montpellier, Montpellier, France; 2 Faculty of Pharmacy, University of Montreal, Montreal, QC Canada; 3 Market Access Department, Lundbeck SAS, Issy-Les-Moulineaux, France; 4 Global Outcomes Research Division, Lundbeck SAS, 43-45, Quai du Président Roosevelt, 92445 Issy-Les-Moulineaux, France

**Keywords:** Alzheimer’s disease, Cholinesterase inhibitors, Memantine, Cost-effectiveness, Nursing home admission, D61, I10, I12, I18

## Abstract

The costs associated with the care of Alzheimer’s disease patients are very high, particularly those associated with nursing home placement. The combination of a cholinesterase inhibitor (ChEI) and memantine has been shown to significantly delay admission to nursing homes as compared to treatment with a ChEI alone. The objective of this cost-effectiveness analysis was to evaluate the economic impact of the concomitant use of memantine and ChEI compared to ChEI alone. Markov modelling was used in order to simulate transitions over time among three discrete health states (non-institutionalised, institutionalised and deceased). Transition probabilities were obtained from observational studies and French national statistics, utilities from a previous US survey and costs from French national statistics. The analysis was conducted from societal and healthcare system perspectives. Mean time to nursing home admission was 4.57 years for ChEIs alone and 5.54 years for combination therapy, corresponding to 0.98 additional years, corresponding to a gain in quality adjusted life years (QALYs) of 0.25. From a healthcare system perspective, overall costs were €98,609 for ChEIs alone and €90,268 for combination therapy, representing cost savings of €8,341. From a societal perspective, overall costs were €122,039 and €118,721, respectively, representing cost savings of €3,318. Deterministic and probabilistic (Monte Carlo simulations) sensitivity analyses indicated that combination therapy would be the dominant strategy in most scenarios. In conclusion, combination therapy with memantine and a ChEI is a cost-saving alternative compared to ChEI alone as it is associated with lower cost and increased QALYs from both a societal and a healthcare perspective.

## Introduction

Alzheimer’s disease (AD) is an irreversible, degenerative brain disease characterised by progressive cognitive dysfunction, associated with behavioural and neuropsychiatric disturbances and functional disability. It is the most prevalent neurodegenerative disease and the most frequent cause of dementia in elderly populations [1]. The prevalence of AD is growing because of the increased longevity of the general populations [2]. It has been estimated that, worldwide, around 35.6 million people have AD, and this figure is expected to increase to 65.7 million by 2030 and 115.4 million by 2050 [3]. A long-term prospective longitudinal cohort study (PAQUID) of the ageing population estimated the prevalence of AD in France to be 4.3 %, doubling with approximately every 5 extra years of age, with an incidence of 1.17 per 100 person years [4]. The total number of cases of AD in France in 2006 has been estimated to be around 900,000 [5].

The costs associated with the care of AD patients are very high, with an estimated per capita annual total cost in the EU of €22,000 in 2008 [6]. This corresponds to a potential total annual cost of €160 billion. In a multinational study of the economic burden of Alzheimer’s disease in 2007 [7], the total cost of all-cause dementia in France was €24 billion, of which informal care costs (€13.5 billion) and nursing home care (€9 billion) were the largest contributors. In addition, patients with dementia account for around one half of all nursing home residents in France [8]. For this reason, delaying nursing home admission may have an impact on the overall costs associated with the disease [9]. The principal factors driving nursing home admission are severity of cognitive impairment, dependency in activities of daily living, and prominent behavioural and psychological symptoms of dementia (BPSD) [10, 11], and treatments ameliorating these functions may be of interest in this respect.

Approved treatments for AD in Europe consist of cholinesterase inhibitors (ChEI) such as donepezil, rivastigmine and galantamine [12] for mild-to-moderate AD and the NMDA receptor antagonist memantine [13, 14] for moderate-to-severe AD. The evidence for the efficacy of memantine in slowing the clinical progression of AD, in improving cognition, memory, communication and the ability to perform daily activities has been reviewed recently [15]. The ability of memantine to decrease symptomatic decline and to improve functioning in patients with moderately severe-to-severe AD was originally demonstrated in two pivotal phase III trials [16, 17] and has subsequently been confirmed in other studies. A meta-analysis of available data published in 2007 concluded that memantine had clinically relevant efficacy in patients with moderate-to-severe AD [18]. In addition, a beneficial effect has also been demonstrated on BPSD [19]. It was subsequently shown that memantine, when given in combination with a ChEI, provided added benefit over ChEIs alone [20]. There is also some evidence that early treatment initiation may increase the chances of an adequate treatment response to memantine [15].

Since the disease features influenced by memantine are important predictors of nursing home admission, it is possible that early treatment with this drug may delay admission to nursing homes. Indeed, a randomised clinical trial comparing memantine to placebo in patients with AD living in the community has provided evidence for a delay in time to nursing home admission and a reduction in caregiver time in patients treated with memantine [21]. Subsequently, an observational study showed that the combination of a ChEI and memantine significantly delayed admission to nursing homes as compared to treatment with a ChEI alone [9].

A cost-effectiveness study published in 2001 using the Assessment of Health Economics in Alzheimer’s Disease (AHEAD) model [22] suggested that the costs of early treatment with a ChEI could be largely offset by a reduction in nursing home costs associated with delayed admission [23]. A more recent pharmacoeconomic modelling study performed in the context of the Canadian healthcare system suggests that this may also be the case for combination therapy with memantine and ChEI, which is more cost-effective than treatment with ChEIs alone because of reduced nursing home costs [24]. However, before drawing a general conclusion from only one study, it is important to ensure that the same health outcomes and economic benefits associated with concomitant memantine and ChEI use compared to ChEI alone with regard to time to nursing home admission can be expected in the context of other healthcare systems, where treatment costs, reimbursement rules and attitudes to nursing home care may vary considerably. For this reason, we have conducted the present analysis in the French healthcare context. The research question addressed was whether use of combination therapy with memantine and ChEI would be cost-effective at standard willingness-to-pay thresholds from both societal and healthcare system perspectives.

## Methods

A cost-effectiveness analysis was conducted to evaluate the economic impact of the concomitant use of memantine and ChEI compared to ChEI alone on the time to nursing home admission. Markov modelling was used in order to simulate transitions over time between discrete health states, each associated with different unit costs. The evaluation was undertaken from a societal perspective and also from the publicly funded healthcare system perspective. For this cost-effectiveness analysis, treatment with ChEI monotherapy was considered the reference care paradigm. The three ChEIs licensed and reimbursed for the treatment of AD in France are donepezil, rivastigmine and galantamine. This evaluation was conducted in compliance with the French guidelines available at the time of the study (CES guidelines for the Economic Evaluation of Health Technologies in France) [25].

### Markov model

**Fig. 1:**
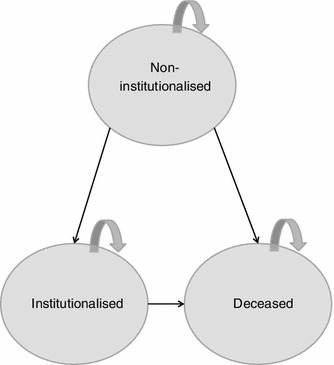
Structure of the Markov model

The Markov model considered three states. These were non-institutionalised (not admitted to a nursing home), institutionalised (admitted to a nursing home) and deceased (Fig. 1). The cohort simulated patients living in the community starting treatment for AD and who had not previously been admitted to a nursing home. The age and gender distributions of the study population were derived from the study by Lopez et al. [9], in which the ChEI monotherapy group had an average age of 76 years old and in which 68.5 % of participants were women. All patients were thus in the non-institutionalised state initially, from where they could move to one of the other two states or remain in the non-institutionalised state at the end of each cycle. It was considered that once patients had entered a nursing home, they would not leave it again. This assumption is supported by a recent survey of nursing home residents in France, of whom less than 1 % return home over a 3-month period [26]. Each cycle corresponded to 1 year and the cohort was simulated over 7 years. This time horizon was chosen since it corresponds to that used in the clinical study of Lopez et al. [9], which was used to generate the transition probabilities for nursing home admissions. In addition, it encompasses the median survival time of AD patients observed in the PAQUID study and thus should be sufficient to capture all relevant benefits of treatment. The structure of the model was identical to that used in the previous Canadian cost-effectiveness study [24].

### Transition probabilities

The probabilities of transition from the non-institutionalised state to the institutionalised state were estimated from data in the Lopez study [9] (Table 1). These transition probabilities differed between the ChEI alone and combination therapy treatment groups, and varied with time across the seven yearly cycles of the model. The transition probability from the non-institutionalised state to death was estimated from all-cause mortality data taken from French mortality tables [27] and adjusted for specific AD-related mortality and for the age and gender distribution of the sample (calculations not shown, available from authors on request). The transition probability between the institutionalised state to death was assumed to be identical to that from the non-institutionalised state to death considered to be independent of treatment. Assuming a different (most likely higher) mortality for institutionalised patients compared to non-institutionalised ones would have led, because of the model structure, to a spurious indirect impact of treatment on life expectancy (through the impact of treatment on institutionalisation). No impact of combination treatment on mortality having been actually observed in the study by Lopez et al. [9], it was therefore decided to use the same death probabilities for institutionalised and non-institutionalised patients in the model. The transition probabilities used in the model are presented in Table 1.

**Table 1:** Transition probabilities

Cycle (years in the model)	Probability of institutionalisation^a^		Probability of death^b^
	ChEI alone	Combination therapy	Both groups
One	0.0167	0	0.0300
Two	0.1031	0	0.0335
Three	0.0947	0	0.0375
Four	0.0808	0.0167	0.0421
Five	0.0891	0.0418	0.0475
Six	0	0	0.0539
Seven	0	0	0.0612

^a^Data were extracted from Fig. 3 of the Lopez et al. publication [9] using Grafula 3 version 2.10 software

^b^French survival tables adjusted for death specifically related to AD (calculations not shown, available from authors on request)

### Utility values

The utility values associated with the institutionalised and non-institutionalised states were the same as those used in an application of the AHEAD model to early treatment with galantamine [23]. However, it should be noted that the ‘non-institutionalised’ and ‘institutionalised’ states used in our model are not strictly identical to the ‘no full time care’ and ‘full time care’ states of the AHEAD model. The utility values were originally derived from a large survey of patients and carers conducted in the US in the 1990s [28], in which utilities were estimated for different levels of disease severity using the Health Utilities Index Mark II, a generic preference index based on the standard gamble. We applied the mean utility score for patients with mild and moderate disease (0.60) to non-institutionalised patients and the mean utility score for patients with severe, profound or terminal disease (0.34) to institutionalised patients. These values were used as weights to calculate QALYs.

### Costs

The costs considered in the analysis included the costs of medication (ChEI and ChEI plus memantine) and also the costs associated with care provided either in the community or in nursing homes. Medication costs were assigned from unit costs reimbursed by the CNAMTS (French national health insurance) [29]. The same unit costs were applied to both nursing home and community care. Regarding the cost of care in either the community or nursing homes, they were obtained from a 2005 national report of the French Assembly on AD [30] and were updated to 2010 levels after the application of the inflation rate using OECD 2005–2010 consumer price inflation rate (Table 2).

**Table 2:** Annual costs

Description	2005 value (€)	2010 value (€)
Costs of medication		
Cholinesterase inhibitors	–	855
Memantine	–	1,158
Costs of community care^a^	17,104	18,757
Direct costs (excluding medication)		
Medical visits	285	313
Hospitalisations	185	203
Nurses	3,326	3,647
Dependency assistance	5,088	5,580
Family contributions	768	842
Other financial aids	2,772	3,040
Indirect costs		
Informal help	4,680	5,132
Costs of nursing home care^a^	26,301	28,843
Direct costs (excluding medication)		
Medical visits	285	313
Hospitalisations	184	202
Care fee	6,560	7,194
Dependency fee	4,872	5,343
Accommodation fee	14,400	15,792

Cost categories correspond to those provided in the 2005 national report of the French Assembly on AD [30]

^a^Original costs are 2005 and were updated to 2010 levels based using OECD 2005–2010 consumer price inflation rate

The costs considered in the analysis including costs of medication (ChEI and ChEI plus memantine) as well as costs associated with care either in the community or in a nursing home are summarised in Table 3.

**Table 3:** Direct and indirect costs per patient in each transition state

Annual costs/patient (€)	State					
	Non-institutionalised		Institutionalised		Death	
	ChEI	ChEI + MEM	ChEI	ChEI + MEM	ChEI	ChEI + MEM
Medications	855	2,013	855	2,013	0	0
Other direct costs	13,625	13,625	28,843	28,843	0	0
Indirect costs	5,132	5,132	0	0	0	0

### Benefit measures and valuation

The primary outcome measure for the evaluation was the incremental cost-effectiveness ratio (ICER), expressed as cost per quality-adjusted life years (QALY) gained. As recommended by the CES [25], the estimated costs and QALYs were discounted at a rate of 0, 3 and 5 %. The base case analysis was discounted at 3 % and discount rates of 0 and 5 % were applied to estimated costs and QALYs in supportive analyses. Half-cycle corrections were performed where required.

### Sensitivity analyses

Deterministic sensitivity and probabilistic analyses were carried out to assess the robustness of the model.

For the deterministic approach, arbitrary variations of ±20 % were applied to the cost and utility parameters (Table 4) and of 50 to 200 % to the transition probabilities (Tables 5, 6). As these variations do not have any impact on transition probabilities equal to zero (probabilities of institutionalisation years 6–7 for the ChEIs alone group and years 1–2–3–6–7), probabilities were also smoothed in order to explore the impact of these null probabilities further. Smoothing of transition probabilities was performed by considering the proportion of patients who were institutionalised at the end of the observation period (7 years), and assuming a constant hazard (i.e. exponential survival model) over the preceding years. For the deterministic sensitivity analyses, two different willingness-to-pay (WTP) thresholds were used, based on current NICE guidelines [31] of £20,000/QALY (€23,065/QALY) for the lower threshold and £30,000/QALY (€34,598/QALY) for the upper threshold.

**Table 4:** Scenarios used in the deterministic sensitivity analysis (costs and utility parameters)

Analysis	Parameter	Base value	Variation (compared to base case) (%)	Range of values	Scenario number	Distribution^b^
Base-case					1	
Costs of community	Direct costs^a^	13,625	80	10,900	2	Triangular
			120	16,350	3	Triangular
	Indirect costs	5,132	80	4,106	4	Triangular
			120	6,159	5	Triangular
Cost of institution	Direct costs^a^	28,843	80	23,074	6	Triangular
			120	34,611	7	Triangular
Utilities	Community	0.60	80	0.48	8	Triangular
			120	0.72	9	Triangular
	Institution	0.34	80	0.27	10	Triangular
			120	0.41	11	Triangular

^a^No sensitivity analysis was conducted on treatment costs as there is no uncertainty related to this parameter

^b^Distributions used for the probabilistic analysis

**Table 5:** Scenarios used in the deterministic sensitivity analysis (transition probabilities–institutionalisation)

Probabilities of institutionalisation												
Years	Base case scenario		50 %		200 %		Worst case scenario (A)		Best case scenario (B)		Smoothed scenario (C)	
	ChEI	ChEI + MEM	ChEI	ChEI + MEM	ChEI	ChEI + MEM	ChEI	ChEI + MEM	ChEI	ChEI + MEM	ChEI	ChEI + MEM
1	0.0167	0.0000	0.0084	0.0000	0.0334	0.0000	0.0084	0.0000	0.0334	0.0000	0.0559	0.0085
2	0.1031	0.0000	0.0516	0.0000	0.2062	0.0000	0.0516	0.0000	0.2062	0.0000	0.0559	0.0085
3	0.0947	0.0000	0.0474	0.0000	0.1894	0.0000	0.0474	0.0000	0.1894	0.0000	0.0559	0.0085
4	0.0808	0.0167	0.0404	0.0084	0.1616	0.0334	0.0404	0.0334	0.1616	0.0084	0.0559	0.0085
5	0.0891	0.0418	0.0446	0.0209	0.1782	0.0836	0.0446	0.0836	0.1782	0.0209	0.0559	0.0085
6	0.0000	0.0000	0.0000	0.0000	0.0000	0.0000	0.0000	0.0000	0.0000	0.0000	0.0559	0.0085
7	0.0000	0.0000	0.0000	0.0000	0.0000	0.0000	0.0000	0.0000	0.0000	0.0000	0.0559	0.0085
Scenario			12		13		14		15		16	

(A) Worst case scenario: the probability of institutionalisation when taking a ChEI alone was reduced by half, whereas the probability associated with taking the combination was doubled; (B) best case scenario: the probability of institutionalisation when taking a ChEI alone was doubled, whereas the probability when taking the combination was reduced by half; (C) smoothed scenario: probabilities of institutionalisation were smoothed assuming a constant hazard over time (i.e. exponential model) in order to explore further the impact of null transition probabilities from the base case scenario

**Table 6:** Scenarios used in the deterministic sensitivity analysis (transition probabilities–death)

Years	Probabilities of death		
	Base-case scenario	50 %	200 %
1	0.0300	0.0150	0.0600
2	0.0335	0.0167	0.0670
3	0.0375	0.0187	0.0749
4	0.0421	0.0210	0.0842
5	0.0475	0.0238	0.0951
6	0.0539	0.0269	0.1078
7	0.0612	0.0306	0.1225
Scenario		17	18

A probabilistic sensitivity analysis was also performed on all the variables using the Monte Carlo method. A total of 10,000 trials were run for the Monte Carlo simulation according to the assigned probability distributions. A triangular distribution was used for all cost and utility parameters. A triangular distribution was also used for the probability of institutionalisation over time. Upper and lower bounds for the probability of institutionalisation were taken from the best and worst case scenarios.

All deterministic and probabilistic sensitivity analyses were conducted from both a publicly funded healthcare system and a societal perspective. The former took into account only direct medical costs and the latter all direct and indirect costs.

### Data analysis and quality control

This economic evaluation was performed using Microsoft Office Excel 2007. The probabilistic sensitivity analyses were performed using Crystal Ball V11.1.2.3. To validate the results, all the analysis components were independently reviewed by two researchers (Catherine Beauchemin and Jean Lachaine, Montreal Pharmacy University). Grafula 3 version 2.10 was used to extract the survival curve data.

## Results

### Cost effectiveness analysis

Over the 7-year time horizon, the mean time to nursing home admission was 4.57 years for patients receiving ChEIs alone and 5.54 years for those receiving combination therapy, corresponding to 0.98 additional years (approximately 1 year) spent in the community for patients receiving combination therapy. This translated into 3.11 QALYs for ChEIs alone and 3.37 QALYs for combination therapy, representing a gain in QALYs of 0.25.

From a healthcare system perspective, overall costs were €98,609 for ChEIs alone and €90,268 for combination therapy, representing a cost saving of €8,341. From a societal perspective, overall costs were €122,039 for ChEIs alone and €118,721 for combination therapy, representing a cost saving of €3,318. From both perspectives, combination therapy is thus a cost-saving and dominant strategy compared to ChEI alone (Table 7). This analysis was performed at a 3 % discount rate. When applying discount rates of 0 and 5 %, combination therapy was consistently dominant over ChEIs alone at both discount rates and from both perspectives (data not shown).

**Table 7:** Incremental cost-effectiveness analysis (base case)

	Survival (years)	Survival at 7 years (%)	Time in community (years)	QALYs	Healthcare perspective	Societal perspective
					Costs (€)	Costs (€)
ChEI alone	5.66	63 %	4.57	3.11	98,609	122,039
ChEI + memantine	5.66	63 %	5.54	3.37	90,268	118,721
Incremental	0		0.98	0.25	−8,341	−3,318
ICER	–	–	–	–	Dominant	Dominant

### Sensitivity analyses

The scenarios tested in the deterministic sensitivity analyses are described in Tables 4, 5, 6. The results of these analyses confirmed the results of the cost-effectiveness analysis for the base case, with combination therapy remaining the dominant alternative in most scenarios.

**Fig. 2:**
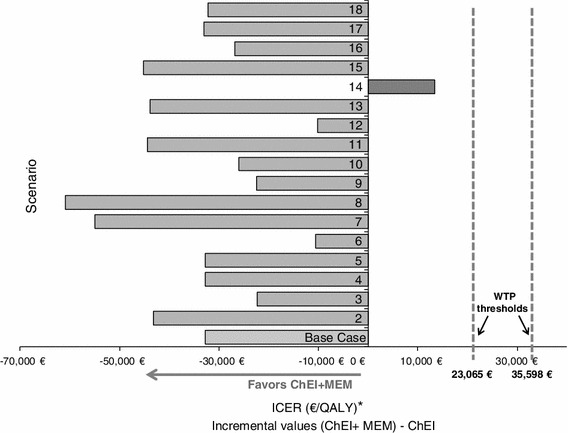
Deterministic sensitivity analysis (healthcare perspective). * All scenarios resulted in a positive incremental effectiveness gain for combination therapy

From a healthcare system perspective, combination therapy remained dominant over ChEI alone in 17 out of 18 scenarios (Fig. 2). The one situation where combination therapy was not dominant was when the worst case scenario for the probability of institutionalisation was applied (scenario 14). In this case, the ICER for combination therapy was 13,342 €/QALY, which could nevertheless be considered cost-effective at willingness to pay thresholds of both €23,065 and €34,598.

**Fig. 3:**
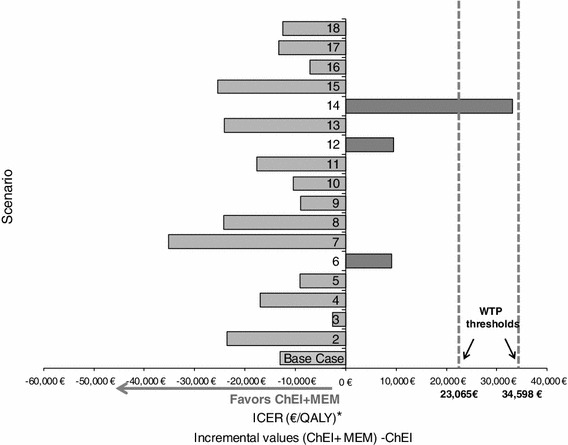
Deterministic sensitivity analysis (societal perspective). * All scenarios resulted in a positive incremental effectiveness gain for combination therapy

From a societal perspective, combination therapy was dominant over ChEI alone in 15 out of 18 scenarios. For the other three scenarios, the combination therapy was deemed cost-effective compared to ChEI alone at the higher willingness-to-pay threshold of €34,598. Two of these scenarios (scenario 6: ICER 9,146 €/QALY; scenario 12: ICER 9,529 €/QALY) remained cost-effective at the lower willingness-to-pay threshold of €23,065, but the combination was no longer deemed cost-effective for scenario 14 (ICER: 33,082 €/QALY; Fig. 3).

A probabilistic sensitivity analysis was also performed using Monte Carlo simulations. From a public healthcare system perspective, combination therapy was a dominant strategy in 99.5 % of the Monte Carlo simulations and was cost-effective in 99.9 % of simulations with WTP thresholds of both €23,065 and €34,598. From a societal perspective, combination therapy was a dominant strategy in 87.0 % of the Monte Carlo simulations and was cost-effective in 99.7 % (lower WTP threshold) and 99.8 % (upper WTP threshold) of simulations.

## Discussion

This cost-effectiveness analysis evaluated the economic consequences of two different treatment options for management of patients with AD living in the community, taking into account the impact on nursing home admission in the French setting. Compared to ChEIs alone, adding memantine to treatment increased the number of QALYs gained and decreased costs due to reduced time spent in nursing homes. Therefore, combination therapy with memantine and a ChEI is a cost-saving alternative compared to ChEI alone, as it is associated with lower costs and increased QALYs from both societal and healthcare perspectives.

These results are consistent with those found in a previous application of the same model to the Canadian setting [24] and with findings of a different model performed in a US setting, in which decline in cognitive function was simulated over 1 year, and the combination of memantine with ChEIs was also found to be cost-effective with respect to ChEIs alone [32]. In all these countries, the overall management costs of Alzheimer’s disease can be significantly reduced by combining memantine treatment with acetylcholinesterase inhibitors, irrespective of the model used, which supports the robustness of the findings obtained. This is important, since it has previously been reported that the Markov model used in the pharmacoeconomic evaluation from the 2007 NICE evaluation [33] was unstable and very sensitive to small changes in input parameters [34].

An important assumption made in our model was that treatments would be used continuously over a lifetime. However, this is unlikely to be the case, since treatment is often discontinued in nursing homes as the disease progresses. The source data that we used for the impact of treatment on institutionalisation was extracted from an observational study of real-life conditions of treatment use [9]. In this study, patients did not in fact receive memantine for the whole 7-year study follow-up (mean treatment duration: 19.2 months). As a consequence, the lifetime treatment assumed in the model does not lead to an overestimation of effectiveness. On the other hand, the cost of treatment is applied for as long as patients are alive and is thus overestimated. The lifetime treatment assumption is consequently a conservative one. We also assumed that medication adherence would be complete in the community setting, whereas observational studies have indicated that adherence of patients with AD to antidementia drugs is suboptimal [35]. Adherence was not documented in the study that was the source of our data. However, again, if adherence is overestimated, this would artifactually inflate treatment costs, resulting in a conservative estimate of cost-effectiveness.

Strengths of this model were that transition probabilities for nursing home admissions were based on real-life data for time to nursing home admission in the two treatment strategies [9] rather than on simulations. These data come from a prospective observational study that is more relevant for cost-effectiveness analysis than data from experimental settings such as randomised clinical trials. Moreover, that costs of care were identified from a recent and relevant official parliamentary report [30]. In addition, the model was iterated over a 7-year time horizon, using measured rather than extrapolated data to determine the transition probabilities. This time horizon is consistent with the expected survival of patients with AD [36]. Finally, an extensive range of sensitivity analyses was performed with regard to the different input parameters, in which combination therapy was consistently found to be cost-effective over ChEIs alone, suggesting that the findings should be considered to be robust.

This study also has several limitations. An important limitation is that local data were not available for all the input variables of the model. Whereas recent French data exist for mortality and costs, there are no local long-term epidemiological data available for time to admission to nursing homes, and we were obliged to use data from a study performed in the USA [9]. Given potential differences in the healthcare systems between France and the USA, the relevance of these US data to the French context is unknown. Similarly, there are no available French data on utilities, and we again used data from a North American study. However, even if there may be issues with transposability, this was addressed in the sensitivity analyses, which demonstrated that the findings were robust and were not altered when utility values and nursing home placement probabilities were modified. Other limitations reflect the limitations of the observational study used as the source for the data on institutionalisation. These have been discussed in detail elsewhere [9] and include lack of randomisation and the subsequent imbalance between the two groups with respect to prognostic risk factors, temporal variation in factors other than treatment that affect institutionalisation and mortality, and a potential Hawthorne effect due to better care provision in the context of the study.

In addition to providing cost savings, as demonstrated by the present cost-effectiveness analysis, delaying admission to nursing homes by providing combination treatment with memantine and ChEI may also be beneficial in terms of patient well-being. A survey of caregivers indicated that admission of AD patients to nursing homes is considered to be a major negative determinant of their quality of life, with more than two-thirds of AD caregivers rating delaying admission as an ‘extremely important’ or ‘very important’ way of maintaining quality of life [37]. Keeping the AD patient at home for as long as possible has thus been identified as a goal for both patients and caregivers [37, 38] and providing support to caregivers identified as a useful strategy to increase the time that AD patients can remain at home [39]. Building such caregiver support is one of the principal strategic axes of the Alzheimer Plan developed in 2008 by the French health authorities to optimise the management of AD patients and to improve the quality of life of both AD patients and their caregivers [40]. In parallel to this, adequate antidementia treatment, and in particular the combination of memantine with ChEIs, could also contribute to allowing AD patients to be managed longer in the community setting [9, 15, 21, 41] and thus help preserve their quality of life.

In conclusion, this economic evaluation indicates that, from both healthcare system and societal perspectives, combination therapy with memantine and a ChEI is a cost-saving strategy in the management of AD patients compared with ChEIs alone.
